# Low-density tissue scaffold imaging by synchrotron radiation propagation-based imaging computed tomography with helical acquisition mode

**DOI:** 10.1107/S1600577523000772

**Published:** 2023-02-16

**Authors:** Xiaoman Duan, Naitao Li, David M. L. Cooper, Xiao Fan Ding, Xiongbiao Chen, Ning Zhu

**Affiliations:** a Division of Biomedical Engineering, College of Engineering, University of Saskatchewan, Saskatoon, SK S7N 5A9, Canada; b Department of Anatomy, Physiology and Pharmacology, College of Medicine, University of Saskatchewan, Saskatoon, SK S7N 5A9, Canada; c Department of Mechanical Engineering, College of Engineering, University of Saskatchewan, Saskatoon, SK S7N 5A9, Canada; d Department of Chemical and Biological Engineering, College of Engineering, University of Saskatchewan, Saskatoon, SK S7N 5A9, Canada; e Canadian Light Source, Saskatoon, SK S7N 2V3, Canada; Australian Synchrotron, Australia

**Keywords:** tissue hydro­gel scaffolds, propagation-based imaging, helical acquisition mode, ring artifact removal

## Abstract

Visualization of low-density tissue scaffolds made from hydro­gels is important yet challenging in tissue engineering and regenerative medicine (TERM). For this, synchrotron radiation propagation-based imaging computed tomography (SR-PBI-CT) has great potential, but is limited due to the ring artifacts commonly observed in SR-PBI-CT images. To address this issue, this study focuses on the integration of SR-PBI-CT and helical acquisition mode (*i.e.* SR-PBI-HCT) to visualize hydro­gel scaffolds. The influence of key imaging parameters on the image quality of hydro­gel scaffolds was investigated, including the helical pitch (*p*), photon energy (*E*) and the number of acquisition projections per rotation/revolution (*N*_p_), and, on this basis, those parameters were optimized to improve image quality and to reduce noise level and artifacts. The results illustrate that SR-PBI-HCT imaging shows impressive advantages in avoiding ring artifacts with *p* = 1.5, *E* = 30 keV and *N*_p_ = 500 for the visualization of hydro­gel scaffolds *in vitro*. Furthermore, the results also demonstrate that hydro­gel scaffolds can be visualized using SR-PBI-HCT with good contrast while at a low radiation dose, *i.e.* 342 mGy (voxel size of 26 µm, suitable for *in vivo* imaging). This paper presents a systematic study on hydro­gel scaffold imaging using SR-PBI-HCT and the results reveal that SR-PBI-HCT is a powerful tool for visualizing and characterizing low-density scaffolds with a high image quality *in vitro*. This work represents a significant advance toward the non-invasive *in vivo* visualization and characterization of hydro­gel scaffolds at a suitable radiation dose.

## Abstract 2

This work shows that combining helical acquisition mode with synchrotron radiation propagation-based imaging computed tomography is a powerful tool for tissue engineering applications with image qualities of high contrast, low noise level and, most importantly, fewer ring artifacts.

## 1. Introduction

In scaffold-based tissue engineering and regenerative medicine (TERM), scaffolds made from biomaterials are used to support and facilitate cell growth and tissue regeneration, as well as transport nutrients and metabolic wastes (Chen, 2019 ▸). For this, scaffolds should possess appropriate architectural, mechanical and biological properties to mimic those of native tissues or organs. As such, visualization and/or characterization of scaffold properties via *in vitro* and/or *in vivo* studies are essential to TERM applications (Duan *et al.*, 2021 ▸). In scaffold-based TERM, hydro­gels are the most commonly used biomaterials for scaffolds with water-swollen crosslinked polymeric networks (Nezhad-Mokhtari *et al.*, 2019 ▸). Typically, hydro­gels have similar density to the surrounding environment (*e.g.* water for *in vitro* study or soft tissue after implantation for *in vivo* study).

To visualize and characterize hydro­gel scaffolds in TERM, synchrotron radiation computed tomography (SR-CT) imaging holds promise for both *in vitro* and *in vivo* applications (Duan *et al.*, 2021 ▸; Zhu *et al.*, 2011 ▸; Ning *et al.*, 2018 ▸, 2021 ▸; Olubamiji *et al.*, 2014 ▸; Naghieh & Chen, 2021 ▸; Izadifar, Babyn, Kelly *et al.*, 2017 ▸; Izadifar, Babyn, Chapman *et al.*, 2017 ▸; Olubamiji *et al.*, 2016 ▸, 2017 ▸; You *et al.*, 2016 ▸; Bawolin & Chen, 2016 ▸). For *in vitro* scaffold visualization, SR-CT is non-destructive/non-invasive imaging which means scaffolds can be imaged without the need for sectioning and other processes. As such, it allows accurate capture of scaffold structures in longitudinal studies of the same scaffolds. It is noted that, among various *in vitro* imaging techniques, scanning electron microscopy (SEM) (Bartoš *et al.*, 2018 ▸; Stachewicz *et al.*, 2019 ▸; Vitas *et al.*, 2019 ▸) has been commonly used for morphology and pore/surface structure of the scaffolds with the wide magnification range 10–500 000× (Zhu *et al.*, 2021 ▸), but the approach involves destructive sample preparation. Such destructive processes may change the structure (*e.g.* pore size) of scaffolds, leading to inaccurate results and findings. Confocal laser scanning microscopy (CLSM) (Bagherzadeh *et al.*, 2013 ▸; Phipps *et al.*, 2012 ▸) is another commonly used technique for tissue scaffold visualization, which enables collection of data in 3D but is limited to the penetration depth range 200–300 µm (Parrilli *et al.*, 2014 ▸). CLSM has been commonly used for quantitative biological analysis and can provide complementary information on tissue scaffolds to SR-CT imaging (morphology). For *in vivo* scaffold visualization, SR-CT has the merits of high spatial resolution and high contrast, as well as a relatively fast scan speed. Magnetic resonance imaging (MRI) (Mueller *et al.*, 2021 ▸; Chen *et al.*, 2020 ▸; Kotecha *et al.*, 2017 ▸), ultrasound imaging (UI), photoacoustic (PA) imaging (Teodori *et al.*, 2017 ▸) and optical coherence tomography (OCT) (Chen *et al.*, 2011 ▸; Wang *et al.*, 2018 ▸) can visualize scaffolds non-destructively and non-invasively. However, MRI requires a long scan time to achieve a high spatial resolution [*e.g.* about 100 h for 100 µm (Edlow *et al.*, 2019 ▸)], whereas SR-CT needs only seconds to minutes to achieve a much higher resolution [*e.g.* lower than 10 µm at dose rates suitable for *in vivo* applications (Harrison *et al.*, 2022 ▸)]. Hydro­gel imaging with a long-time scan may change some of the properties of a hydro­gel, leading to motion artifacts. High-resolution imaging (<100 µm) of UI/PA requires high-frequency scanning, which may heat up scanned scaffolds. Besides, for *in vivo* UI, the presence of bone can greatly limit the resolution. OCT can achieve a high resolution, but its penetration depth is a shortcoming compared with SR-CT, particularly for full scaffold imaging *in vivo* in animal models (*e.g.* rats). Compared with conventional absorption contrast CT imaging using a conventional (polychromatic) X-ray tube as a source, monochromatic SR-CT can obtain both absorption contrast and phase contrast. The latter can achieve a higher contrast for low-density scaffolds, especially when combined with phase retrieval (PhR). It is noted that SR-CT is a broad class of techniques that includes interferometer CT (Miao *et al.*, 2016 ▸), edge-illumination CT (Hagen *et al.*, 2014 ▸; Momose *et al.*, 2003 ▸), diffraction-enhanced imaging CT [SR-DEI-CT (Chapman *et al.*, 1997 ▸)], analyzer-based imaging [SR-ABI-CT (Wernick *et al.*, 2003 ▸)] and propagation-based imaging CT [SR-PBI-CT (Suzuki *et al.*, 2002 ▸)]. SR-PBI-CT has the great advantage of simple implementation and fast acquisition compared with SR-DEI-CT/SR-ABI-CT (Izadifar *et al.*, 2016 ▸). As such, it has great potential for clinical studies (Fedon *et al.*, 2018 ▸; Longo *et al.*, 2014 ▸; Castelli *et al.*, 2011 ▸) and *in vivo* animal imaging (Taba *et al.*, 2018 ▸). Therefore, this work involved the application of SR-PBI-CT to hydro­gel scaffold imaging.

Despite many advantages, SR-PBI-CT image quality for low-density scaffolds still requires improvement. Ring artifacts, for example, are an issue for SR-PBI-CT (Pelt & Parkinson, 2018 ▸) due to systematic errors or defects on the scintillator, monochromator or filters. Such artifacts usually make it difficult to process and analyze images using existing methods for visualizing and charactering samples. This is a particular concern for imaging low-density scaffolds due to their relatively low contrast. Methods have been developed to reduce ring-like artifacts, including pre-processing and post-processing algorithms, but these approaches suffer from various limitations in practical application. For example, low-pass Fourier filtering (Raven, 1998 ▸) poses the risk of introducing additional artifacts in the reconstructed background. The Sarepy sorting wind method (Vo *et al.*, 2018 ▸) usually has limited ring artifact removal efficacy for noisy images and for images with unresponsive stripes which result from dead pixels on the detector and/or damaged areas of the scintillator. In addition, these algorithms involve manual hyperparameter selection (*e.g.* the size of convolution kernel or window size which are defined by the user). Different values will produce different outcomes and it usually takes a long time to determine the optimal value. Besides, for different regions of interest (ROIs), the hyperparameter values need to be finely tuned for optimal artifact removal and therefore subjectivity is a limitation. These limitations raise a great need to address the issue of ring artifacts if SR-PBI-CT is to realize its larger potential for imaging of low-density targets, including hydro­gel scaffolds.

This paper explores the integration of SR-PBI-CT with helical acquisition mode (hereafter SR-PBI-HCT) to address the ring artifact issues which negatively impact hydro­gel scaffold visualization. Helical acquisition mode has been widely used for clinical CT, but is still relatively novel when combined with SR-PBI-CT. To the best of our knowledge, the SR-PBI-HCT technique, though reported previously (Pelt & Parkinson, 2018 ▸), has not yet been used in the visualization and characterization of scaffolds for tissue engineering applications. To optimize image quality of hydro­gel scaffolds with SR-PBI-HCT, we studied the impact of helical pitch (*p*, definition given in Section 2.2), X-ray photon energy (*E*) and the number of acquisition projections per revolution (360°) (*N*_p_). Although some of these have been discussed previously (Oliva *et al.*, 2020 ▸; Taba *et al.*, 2019 ▸; Nesterets *et al.*, 2018 ▸) for regular SR-PBI-CT imaging, optimal parameters for SR-PBI-HCT remain unclear. Furthermore, we evaluated the image quality of hydro­gel scaffolds using SR-PBI-HCT at a low-level radiation dose (*i.e.* 342 mGy, which is acceptable for *in vivo* imaging). Our present study, though carried out *in vitro*, is thus intended to serve as a step towards 3D non-invasive *in vivo* characterization for TERM applications using hydro­gel scaffolds.

## 2. Materials and methods

### 2.1. Scaffold preparation

In this work, 4% *w*/*v* aqueous alginate made from medium-viscosity alginate powder (alginic acid sodium salt from brown algae, CAS 9005–38-3, Sigma–Aldrich) and mixed material solutions (3% *w*/*v* alginate with 1% *w*/*v* gelatin, made from gelatin powder from porcine skin, G1890, Sigma) were prepared. The preparation process was similar to the procedure developed in our previous work (Ning *et al.*, 2021 ▸). The prepared solutions were then magnetically stirred at room temperature overnight, or until thoroughly mixed. The crosslinking solution was a calcium chloride dihydrate (CaCl_2_·2H_2_O, CAS 10035–04–8, Sigma–Aldrich) solution at 50 m*M* concentration, with 0.1% *w*/*v* polyethyl­ene­imine (PEI, J61270, Alfa Aesar) solution used as the solvent. The surfaces of 12-well plates were coated with 3 ml of the same PEI solution and were left in an incubator at 37°C overnight. The PEI coating was used to ensure that the scaffolds did not stick to the well plate once printing and crosslinking were complete.

**Figure 1:**
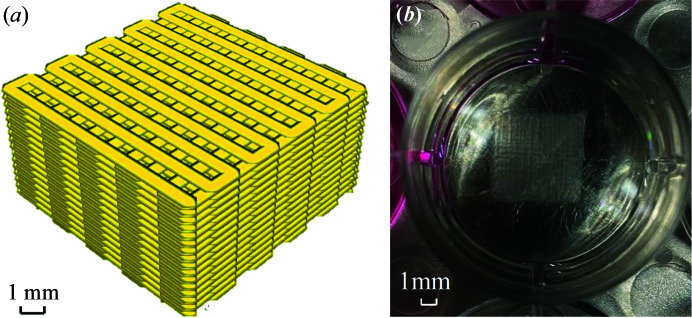
Alginate scaffold: (*a*) designed scaffold structure and (*b*) printed scaffolds.

The next day, the PEI solution coated on printed plates was replaced with 3–4 ml of the crosslinking solution. The syringes containing different solutions were loaded into the bioprinter and attached to the printing arm. An envisionTEC 4th Generation 3D-Bioplotter Manufacturer Series – an extrusion based (pneumatic) bioprinter – was utilized. Scaffolds were fabricated with dimensions 10 mm × 10 mm × 5 mm with a strand diameter of 500 µm following a grid pattern [Fig. 1 ▸(*a*)] with inter-strand distances of 1 mm and 1.5 mm. These were kept in containers in a fridge at 4°C for 2 days.

### 2.2. Synchrotron imaging system setup

**Figure 2:**
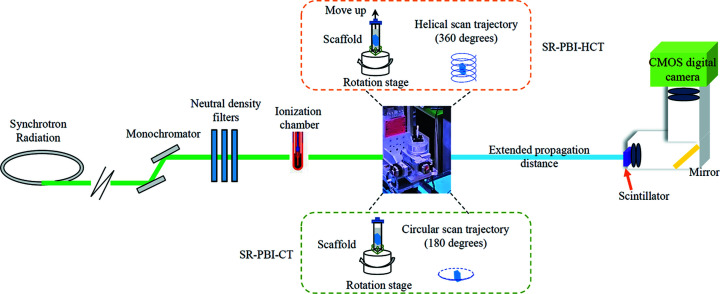
Schematic of the SR-PBI-HCT and SR-PBI-CT imaging setups.

The SR-PBI-HCT imaging experiments were performed at the Biomedical Imaging and Therapy (BMIT) 05ID-2 beamline (Wysokinski *et al.*, 2007 ▸) of the Canadian Light Source. On this beamline, the double-crystal Si(111) monochromator can produce photon energies of 25–140 keV. Fig. 2 ▸ displays a schematic diagram illustrating the SR-PBI-HCT and SR-PBI-CT imaging setups. The essence of PBI is propagation with distance [*i.e.* distance from sample to detector (SDD)], which can turn phase distortions into interference fringes and produce large contrast values at the edges of structures. All scans were performed at SDD = 1.5 m, a distance that can provide satisfactory contrast and spatial resolution for hydro­gel scaffold imaging with a reconstructed voxel size of 13 µm based on a previous study by our group (Section S1 of the supporting information). For parallel beam geometry, SR-PBI-HCT scanning usually involves a horizontal rotation range over 360° for achieving the full imaging of the ROI (Section S2 of the supporting information), whereing SR-PBI-CT scanning takes place with a rotation of exactly 180°. The adjustment of photon flux can be achieved through placing neutral density filters (NDFs) with particular thickness in the beam. The detector, with a pixel size of 13 µm and an image depth of 16 bit, consists of a beamline monitor (AA60 HAMAMATSU, Japan) with a scintillator [LuAg500, lutetium Lu3Al5O12 garnet (LuAG) doped by the luminescent Ce^3+^, thickness of 500 µm] converting X-rays to visible light, an optic system and a complementary metal–oxide semiconductor (CMOS) digital camera (ORCA Flash 4.0). The active areas of the detector for SR-PBI-CT and SR-PBI-HCT imaging are 716 × 2048 pixels (9.31 mm × 26.624 mm) and 200 × 2048 pixels (2.6 mm × 26.624 mm), respectively.

As discussed, *p* is a parameter for SR-PBI-HCT imaging, which can be defined as

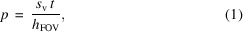

where *s*_v_ is the vertical speed (mm s^−1^) of the rotation stage, *t* is the required time (s) for a full rotation/revolution and *h*_FOV_ is the height of the field of view (FOV) (mm) (*i.e.* the height of the active detector).

In addition, the radiation dose was measured using a calibrated ionization chamber (PTW 31010, Freiburg, Germany). The dose rate 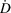 (Gy s^−1^) was measured and then the surface entry radiation dose *D* (Gy) using SR-PBI-HCT was calculated by

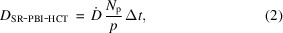

where Δ*t* is the exposure time (s) for each projection and *N*_p_/*p* is the effective projection number per pitch. Since SR-PBI-CT only involves half a revolution (*i.e.* 180°) and there is no pitch, the effective projection number is given by *N*_p_/2. Given the fact that the beam flux may not be exactly uniform (especially for SR-PBI-CT, as seen later in Fig. 5) and that the surface entry dose rate was measured at a position in front of the geometric center of the detector in our study, the dose rate measured is approximately its maximum value and the dose evaluated from equation (2) is the approximate maximum surface entry dose.

### 2.3. Imaging of hydro­gel scaffolds with SR-PBI-HCT

Table 1 ▸ displays the detailed experimental imaging conditions for five different test groups. The image quality of hydro­gel scaffolds using both SR-PBI-HCT and SR-PBI-CT was first evaluated. In addition, in order to determine the spatial resolution of SR-PBI-HCT and SR-PBI-CT, a 3D bar pattern phantom (5 mm × 5 mm, QRM, Möhrendorf, Germany) was also imaged. Then, we conducted three parameter test experiments using SR-PBI-HCT, *i.e.* varied *p* (1.3, 1.5, 1.7, 1.9 and 2.5), *E* (30 keV, 40 keV and 50 keV) and *N*_p_ (500, 1000 and 3000), and examined their corresponding effects on hydro­gel scaffold image quality qualitatively and quantitatively. Due to the limited active detector height, multiple rotations (*i.e.* three rotations for *p* = 1.3, 1.5, 1.7, 1.9, and two rotations for *p* = 2.5) were taken for imaging the scaffolds when using SR-PBI-HCT. In the end, we scanned the hydro­gel scaffold using SR-PBI-HCT at a low-level radiation dose (*i.e.* 342 mGy) to evaluate the feasibility of SR-PBI-HCT for *in vivo* imaging.

### 2.4. Image processing and evaluation metrics

**Figure 3:**
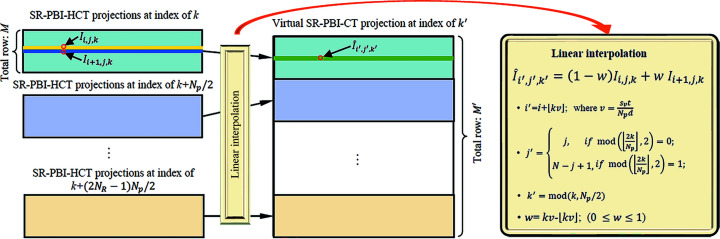
Schematic of virtual projection stitching of SR-PBI-CT from SR-PBI-HCT projections. *I*_*i*,*j*,*k*_ and 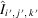 are the gray value of the SR-PBI-HCT projection and the gray value of the converted virtual SR-PBI-CT projection at the positions (*i*, *j*, *k*) and (*i*′, *j*′, *k*′), respectively. *i* and *i*′ are the row indexes, *j* and *j*′ are the column indexes, and *k* and *k*′ are the projection indexes. 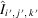 is the linear interpolation result of *I*_*i*, *j*, *k*_ and *I*_*i*+1,*j*,*k*_ with a weight of 1 − *w* and *w*. The virtual SR-PBI-CT projection at an index of *k*′ is transformed from a sequence of SR-PBI-HCT projections at index of *k*, *k* + *N*_p_/2,…, *k* + (2*N*_R_ − 1)*N*_p_/2 with an interval of *N*_p_/2. The total row *M* of the SR-PBI-HCT projection is 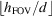 while the total row *M*′ of the virtual SR-PBI-CT projection is 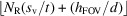 . mod(·,·) denotes the modulo operator.

In order to apply existing image reconstruction and post-processing algorithms tailored to SR-PBI-CT, the projections obtained using SR-PBI-HCT needed to be converted to virtual projections acquired by SR-PBI-CT (Pelt & Parkinson, 2018 ▸; Fu *et al.*, 2014 ▸). The steps to form virtual projections in our study are presented schematically in Fig. 3 ▸ and the Python script is available on GitHub: https://github.com/Xiaoman896/HCT2CT.

Assuming the rotation stage is moved downwards, this conversion process can be expressed by

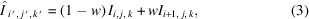

where *i* and *i*′ are the row indexes, *j* and *j*′ are the column indexes, and *k* and *k*′ are projection indexes. *I*_*i*, *j*, *k*_ and 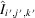 denote the gray value of the SR-PBI-HCT projection and the gray value of the converted virtual SR-PBI-CT projection at the (*i*, *j*, *k*) and (*i*′, *j*′, *k*′) positions, respectively; *w* is the linear interpolation weight.

Values* i* and *i*′ are *i* = 1, 2,…, *M*, where 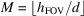 and *i*′ = 1, 2,…, *M*′, and *M*′ = 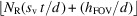 . *N*_R_ is the number of rotations and *d* is the pixel size [*s*_v_, *t* and *h*_FOV_ have the same definitions as in equation (1)]. Let 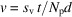 be the vertical movement speed (units of pixels per projection), then the vertical translation for the *k*th projection will be *kv* (unit of pixels). The relationship between *i*′ and *i* can be expressed by

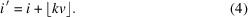

Given the fact that there is no displacement/translation in the horizonal direction for SR-PBI-HCT scanning, we have

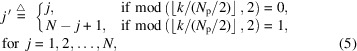

where mod(…) denotes the modulo operator and *N* is the total column number of the SR-PBI-HCT projection. The above equation indicates that the column index needs to be horizontally flipped if 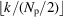 is odd.

*k* and *k*′ are the projection indexes and *k* = 0, 1,…, *N*_*R*_ *N*_p_ − 1 (corresponding angle range from 0 to 360*N*_R_°) while *k*′ = 0, 1,…, *N*_p_/2 − 1 (corresponding angle range from 0 to 180°). The relationship between *k* and *k*′ is given by

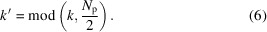


*w* in equation (3) is the linear interpolation weight and can be calculated by

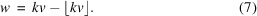

In the present study, we noticed that, if *p* < 2, there were some redundant data (*i.e.* same rows appearing in different projections) for the SR-PBI-HCT projections during conversion, which were discarded.

Before the projection conversion from SR-PBI-HCT to virtual SR-PBI-HCT, the background (*i.e.* flat and dark) corrections were first completed. Then, the transport of intensity equation (TIE) (Paganin *et al.*, 2002 ▸), a popular PhR algorithm, was performed on each projection. The δ/β value (2000 for all cases) can be calculated approximately (Thompson & Vaughan, 2001 ▸). The open-source software package *Ultra-Fast-Online* (*UFO*) was used to perform PhR (*i.e.* TIE) on the projections and the CT reconstruction [filtered-backprojection (FBP) algorithm] (Vogelgesang *et al.*, 2016 ▸). In addition, after PhR, images obtained using SR-PBI-CT were also processed with common ring artifact removal methods, *i.e.* low-pass Fourier filtering (Raven, 1998 ▸) and the Sarepy sorting wind method (Vo *et al.*, 2018 ▸). For low-pass Fourier filtering, the essence of ring artifact removal is to filter the vertical stripes (*i.e.* ring artifact areas) in the 2D frequency domain, and the key parameters that need to be tuned are the horizontal and vertical sigma, *i.e.* 10 and 1 in our work, respectively. For the Sarepy sorting wind method, the essence of ring artifact removal is to employ a median filter (along the horizontal direction) to remove vertical stripes, where the first step is to retrieve the response of each pixel by sorting intensities along each column of a sinogram. The key parameters to be tuned are the window size and the signal-to-noise ratio (SNR). The window size indicates the median filter size and, in our study, a value of 10 (unit of pixels) was selected. The SNR parameter controls the sensitivity of the stripe detection and a value of SNR from 1.1 to 3.0 is recommended. To compare image quality across the different imaging conditions, both objective estimation of quantitative evaluation metrics and subjective evaluation (*e.g.* image perception and cognition) were employed. Quantitative evaluation metrics include SNR and contrast-to-noise ratio (CNR) (Yao *et al.*, 2019 ▸), which are formulated as

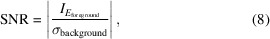




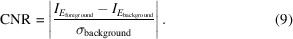


*I*_*E*_ and σ denote the mean gray value and the standard deviation of the ROI, respectively. The foreground represents the ROI including objective hydro­gel scaffolds and the background represents the ROI excluding objective samples.

In addition to SNR and CNR, which mainly focus on the image noise level, the modulation transfer function (MTF) (Fujita *et al.*, 1992 ▸) of the 3D QRM bar pattern phantom CT images and Fourier shell correlation (FSC) (Van Heel & Schatz, 2005 ▸) were also calculated for the spatial resolution analysis. FSC is used to estimate the correlation coefficient between Fourier shells of two 3D reconstructions computed from two independent datasets. In our study, two mutually independent datasets were generated by scanning scaffolds with double projection numbers per rotation and using the odd projections for one reconstruction and the even projections for a second reconstruction.

## 3. Results and discussion

### 3.1. Comparison of SR-PBI-HCT and SR-PBI-CT

**Figure 4:**
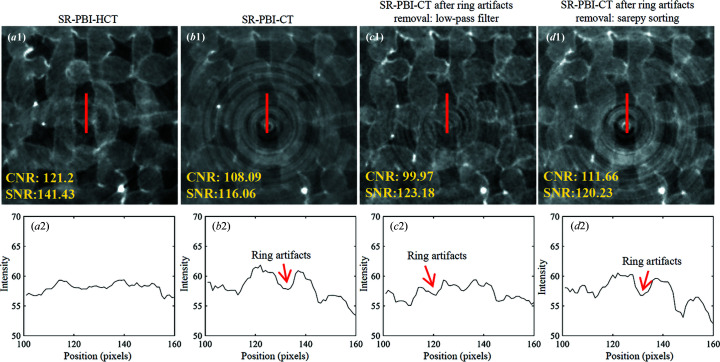
Comparison of SR-PBI-HCT and SR-PBI-CT (gray scale: 0–255); (*a*1) SR-PBI-HCT image, (*b*1) SR-PBI-CT image, (*c*1) SR-PBI-CT image with low-pass Fourier filtering and (*d*1) SR-PBI-CT image with Sarepy sorting. (*a*2)–(*d*2) corresponding gray value profiles of ROIs at red line positions.

The reconstructed hydro­gel scaffold (4% *w*/*v* alginate) images using SR-PBI-HCT and SR-PBI-CT are shown in Fig. 4 ▸. From the results [Figs. 4 ▸(*a*1)–4 ▸(*d*1)], the SR-PBI-HCT image does not show obvious artifacts whereas the SR-PBI-CT image presents serious ring artifacts. Such artifacts disrupt the continuity of the strand grayscale values in the image and thus impair the capacity to accurately visualize/analyze strand properties. Although these artifacts can be removed somewhat using image-processing algorithms, there are still some obvious remaining artifacts. The gray value profiles across strands [Figs. 4 ▸(*a*2)–4 ▸(*d*2)], as indicated by the red lines in Figs. 4 ▸(*a*1)–4 ▸(*d*1), were also visualized, providing the quantitative contrast information.

SR-PBI-HCT can cause helical artifacts due to the nature of its helical acquisition. Compared with ring artifacts, helical artifacts spread over larger regions of the volume and thus have reduced effects. In this work, we applied a linear interpolation in the conversion process from SR-PBI-HCT projections to virtual SR-PBI-CT projections to lessen the effect of defects (*i.e.* the cause of artifacts). As a result, SR-PBI-HCT has significantly reduced artifacts compared with SR-PBI-CT.

**Figure 5:**
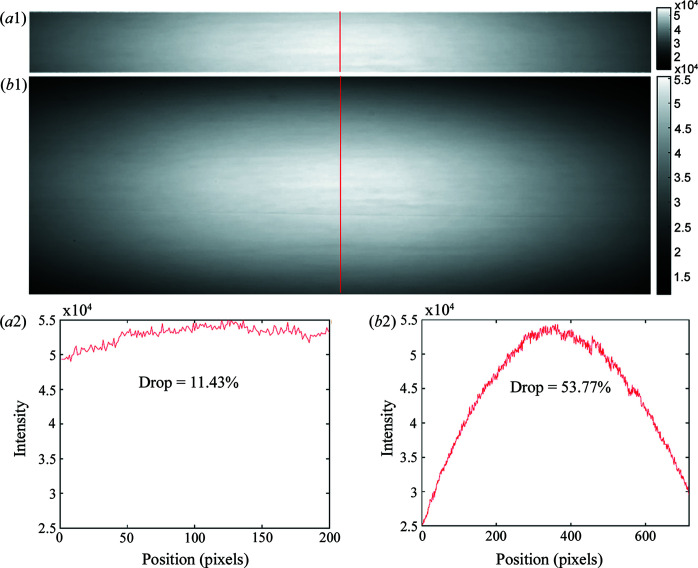
X-ray flux distribution comparison between SR-PBI-HCT and SR-PBI-CT. Flat images of SR-PBI-HCT (*a*1) and SR-PBI-CT (*b*1). Gray value profiles of the flat images (shown by the red lines) of SR-PBI-HCT (*a*2) and SR-PBI-CT (*b*2).

Theoretically, the use of a large-area detector and/or increased pitch would help to further reduce the helical artifacts because these can spread artifacts over a larger region of the volume. However, such settings may lead to other issues, for example, additional artifacts/noise problems if using a large-area detector (see Fig. 5 ▸ and related discussion), and streaking artifacts if using a pitch of more than 2 (see Fig. 8 and related discussion). As such, the imaging settings become complicated, particularly for the case of imaging live animals where the radiation dose is critical; as such, a trade-off has to be made between the radiation dose and imaging quality.

In addition, we noticed that the results from SR-PBI-HCT have higher values of SNR and CNR than those of SR-PBI-CT (even for areas without ring artifacts). One of the reasons behind this could be the different intensity/flux distribution ranges in the vertical direction due to different active detector height (*i.e.* 2.6 mm for SR-PBI-HCT and 9.31 mm for SR-PBI-CT). Fig. 5 ▸ shows the flat-field images and their vertical gray value profiles (*i.e.* flux distribution) obtained using SR-PBI-HCT and SR-PBI-CT.

From Figs. 5 ▸(*a*1) and 5 ▸(*b*1), SR-PBI-HCT only uses the central X-ray beam so that the overall intensity is stronger and the distribution is more uniform compared with the beam received by a larger detector in SR-PBI-CT. Specifically, for SR-PBI-HCT, the vertical X-ray flux intensity drop 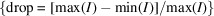 is 11.43% (from center to sides), which is much smaller than the drop of 53.77% for SR-PBI-CT. Although the scanned scaffold has a smaller height (5 mm) than the active height of the detector (9.31 mm), the intensity differences still negatively affect the image quality (*i.e.* increased noise level in the relatively low-flux areas). This effect will become more severe with low-dose imaging for *in vivo* imaging. Generally, the smaller detector in SR-PBI-HCT overcomes the limitation of beam height for some cases and is also beneficial to control radiation dose distribution for *in vivo* imaging. Additionally, the small detector in SR-PBI-HCT reduces the possibility of including damaged pixels, thereby decreasing the possibility of introducing additional unexpected artifacts. Although objects can also be imaged with SR-PBI-CT using a small detector via multiple view scanning (by moving the sample longitudinally for the next scan), the intensity distributions are less uniform because overlapping only occurs at the margins between different views. The overlapping in SR-PBI-CT also increases the radiation dose compared with continuous scan mode in SR-PBI-HCT.

**Figure 6:**
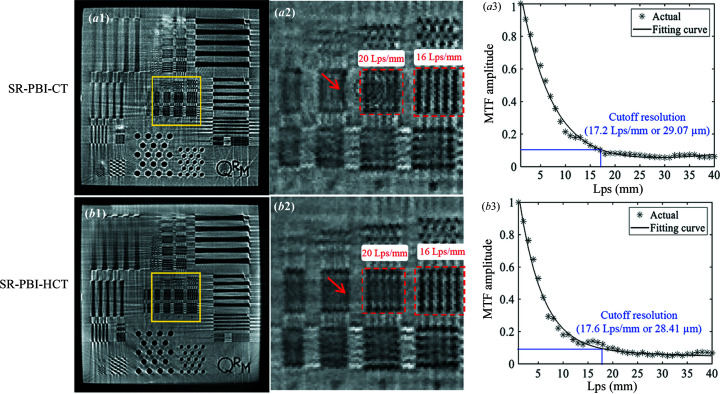
Reconstructed 3D QRM bar pattern phantom using SR-PBI-CT and SR-PBI-HCT (gray scale: 0–255); (*a*1) SR-PBI-CT image and (*b*1) SR-PBI-HCT image and the corresponding (*a*2) SR-PBI-CT and (*b*2) SR-PBI-HCT ROIs enlarged from the yellow squares in (*a*1) and (*b*1); (*a*3)–(*b*3) corresponding MTF amplitudes with cutoff resolutions shown.

Fig. 6 ▸ displays the reconstructed results of the 3D QRM bar pattern phantom from SR-PBI-HCT and SR-PBI-CT for the analysis of spatial resolution. None of the PhR and ring artifact removal algorithms were applied to avoid introducing impacts on spatial resolution. From Figs. 6 ▸(*a*2) and 6 ▸(*b*2), the line pairs at the center region of the phantom show comparable spatial resolution reflected by the similarly discernible line pairs between SR-PBI-HCT and SR-PBI-CT. However, due to the effect of ring artifacts, we notice that there are obvious structural distortions on the line pairs for SR-PBI-CT, as indicated by the red arrows. The curves in Figs. 6 ▸(*a*3) and 6 ▸(*b*3) show the relationship between the MTF amplitude and spatial resolution [measured as line-pairs per millimetre (Lps mm^−1^)], as well as the corresponding cutoff resolution (10% MTF, *i.e.* smallest resolvable object) of 17.2 Lps mm^−1^ (*i.e.* linewidth: 29.07 µm) for SR-PBI-CT and 17.6 Lps mm^−1^ (*i.e.* linewidth: 28.41 µm) for SR-PBI-HCT. SR-PBI-HCT shows a slightly higher spatial resolution than SR-PBI-CT and the main reason may be the disruption of ring artifacts on SR-PBI-CT images. By combining the results from Figs. 4 ▸ and 6 ▸, SR-PBI-HCT shows promise of addressing the ring artifacts, while having no loss in spatial resolution.

### 3.2. Influence of helical pitch of SR-PBI-HCT

**Figure 7:**
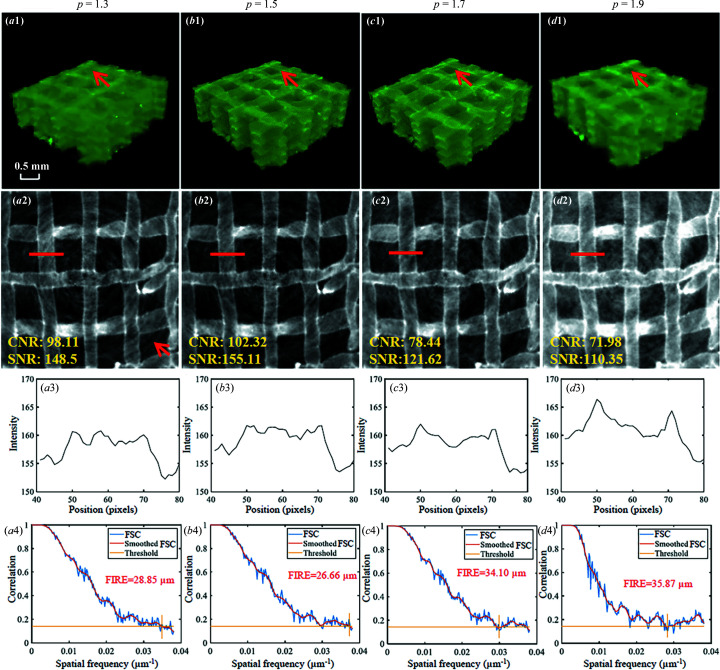
Effect of helical pitches on hydro­gel scaffold images using SR-PBI-HCT with pitches of 1.3, 1.5, 1.7 and 1.9; (*a*1)–(*d*1) reconstructed 3D results; (*a*2)–(*d*2) corresponding 2D images; (*a*3)–(*d*3) corresponding gray value profiles measured at the red line positions in (*a*2)–(*d*2); (*a*4)–(*d*4) 3D spatial resolution analyses with FSC, smoothed FSC and FIRE.

**Figure 8:**
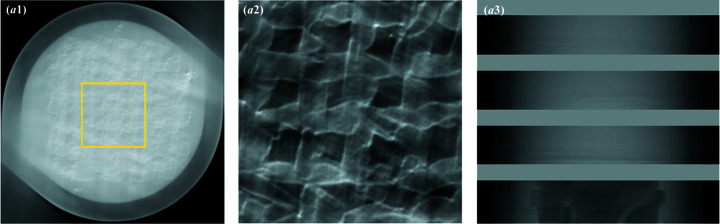
Hydro­gel scaffold images with a pitch of 2.5. (*a*1) Reconstructed slice; (*a*2) ROI [yellow square in (*a*1)]; (*a*3) example of converted virtual projections (gray regions are missing information between adjacent rotations).

The effect of *p* on hydro­gel scaffold images is shown in Fig. 7 ▸. Overall, there is little visible effect on reconstructed 3D results of *p* [Figs. 7 ▸(*a*1)–7(*d*1)] with the range 1.3–1.9, and all the strands within the assessed ROIs are visibly recognizable [Figs. 7 ▸(*a*2)–7(*d*2)]. The gray value profiles of strands for all cases are very close to each other [Figs. 7 ▸(*a*3)–7(*d*3)]. Nevertheless, one can still observe the slight artifacts on images obtained with *p* = 1.3, as indicated by the red arrow [Fig. 7 ▸(*a*2)]. These artifacts are diminished for images with *p* = 1.5, 1.7 and 1.9. This is because higher *p* actually disperses the ring artifacts in a larger vertical region. As a result, the artifacts assigned to each SR-PBI-HCT slice will be reduced. In addition, SNR and CNR are improved when *p* increases from 1.3 to 1.5, but are then reduced when *p* increases from 1.5 to 1.9. The analysis of 3D spatial resolution [Figs. 7 ▸(*a*3)–7(*d*3)] with FSC also produces similar results. The Fourier image resolution (FIRE) is the reciprocal of spatial frequency at the intersection of the smoothed FSC curve with a correlation threshold of 1/7 (*i.e.* 0.14). One possible reason could be, for a reconstructed slice, the illumination range of X-rays (*i.e.* the total angle covered by the flux when samples are rotating) decreases with increasing *p*. A small illumination range usually produces worse image quality (Hayes *et al.*, 2021 ▸). In summary, *p* = 1.5 can produce higher image quality because it balances the effects from ring artifact dispersion and illumination range. For a parallel beam geometry system, it should be realized that, when *p* > 2.0, overlapping between adjacent rotations will be incomplete, and this will lead to streaking artifacts, as shown in Fig. 8 ▸ (*p* = 2.5).

### 3.3. Influence of photon energy on SR-PBI-HCT

**Figure 9:**
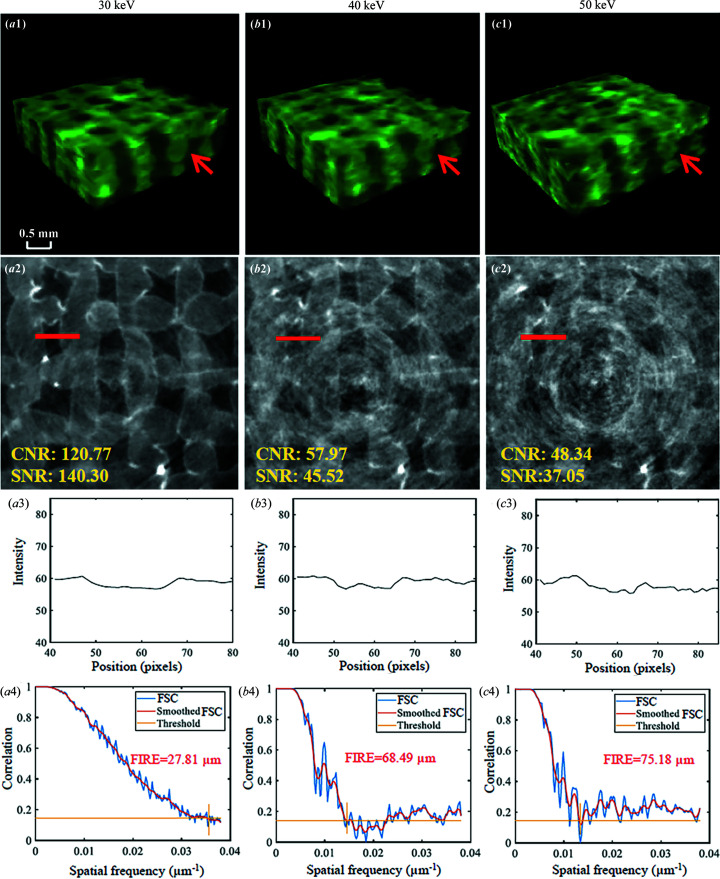
Effect of photon energies on hydro­gel scaffold images using SR-PBI-HCT with 30 keV, 40 keV and 50 keV; (*a*1)–(*c*1) reconstructed 3D results; (*a*2)–(*c*2) corresponding ROI images; (*a*3)–(*c*3) corresponding gray value profiles measured at the red line positions in (*a*2)–(*c*2); (*a*4)–(*c*4) 3D spatial resolution analysis with FSC, smoothed FSC and FIRE.

The effect of *E* on hydro­gel scaffold images using SR-PBI-HCT is shown in Fig. 9 ▸. The main differences associated with X-ray energies are the variations in image contrast, which generally involve the combination of phase contrast and absorption contrast in synchrotron radiation imaging. In the typical X-ray photon energy range 10–140 keV, the absorption coefficient scales as 1/*E* ^3^ (Als-Nielsen & McMorrow, 2011 ▸) before or after the absorption edge. A lower *E* has a higher absorption coefficient and therefore is less penetrating. When *E* = 40 keV and 50 keV, the X-ray photons penetrate with less absorption, resulting in little difference in image contrast (Fig. 9 ▸). Thus, the strands are hard to identify for *E* = 40 keV and 50 keV [Figs. 9 ▸(*b*2) and 9 ▸(*c*2)] and the edges of strands are not clear [Figs. 9 ▸(*b*1) and 9 ▸(*c*1), as indicated by the red arrows]. Besides, 3D spatial resolution analysis with FSC shows that both 40 keV and 50 keV have much larger values of FIRE than 30 keV [Figs. 9 ▸(*b*1) and 9 ▸(*c*1)]. Consequently, to obtain scaffold images with higher SNR and CNR using SR-PBI-HCT, 30 keV is optimal compared with 40 keV and 50 keV. Theoretically, with *E* < 30 keV, imaged objects have a higher absorption coefficient and therefore result in higher contrast; however, for our scaffolds, imaging in a tube with 50 ml water (*in vitro*, simulating *in vivo* imaging conditions for scaffolds implanted in a rat leg for nerve tissue regeneration), *E* < 30 keV results in higher noise level and higher radiation dose because a larger proportion of photons are absorbed.

### 3.4. Influence of projection numbers of SR-PBI-HCT

**Figure 10:**
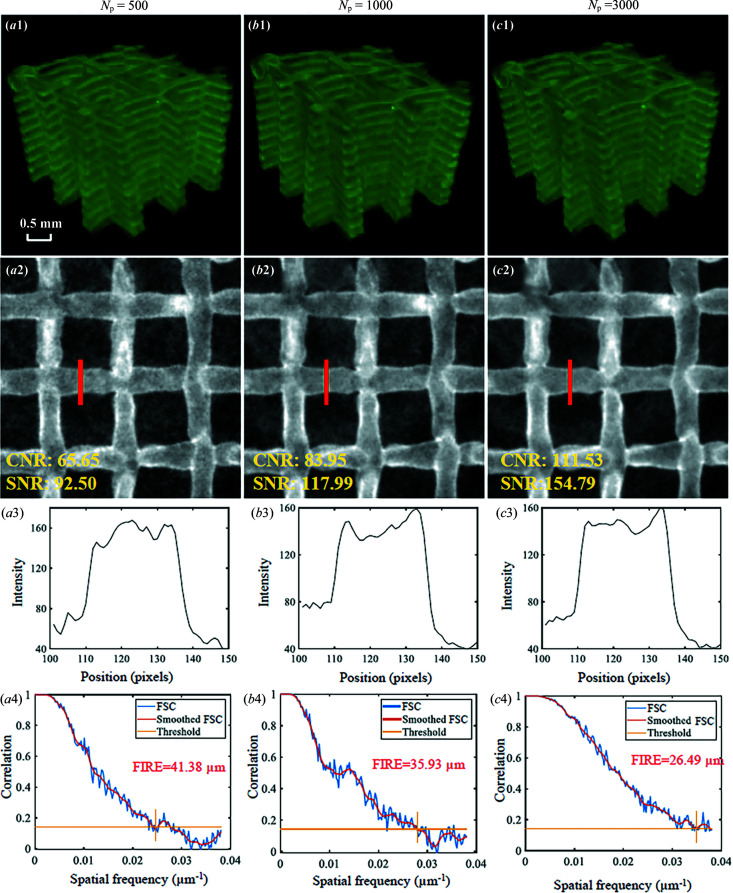
Effect of projection numbers on hydro­gel scaffold images using SR-PBI-HCT with *N*_p_ = 500, 1000 and 3000; (*a*1)–(*c*1) reconstructed 3D results; (*a*2)–(*c*2) corresponding ROI images; (*a*3)–(*c*3) corresponding gray value profiles measured at the red line positions in (*a*2)–(*c*2); (*a*4)–(*c*4) 3D spatial resolution analysis with FSC, smoothed FSC and FIRE.

The effect of *N*_p_ on hydro­gel scaffold images is shown in Fig. 10 ▸. For *N*_p_ = 500, 1000 and 3000, hydro­gel scaffolds present sufficient image contrast for segmentation and 3D volume rendering display [Figs. 10 ▸(*a*1)–10 ▸(*c*1)]. Although the noise is increased for *N*_p_ = 500 and 1000 compared with *N*_p_ = 3000, strands can still be clearly identified [Figs. 10 ▸(*a*2)–10 ▸(*c*2)], which is also supported by quantitative analysis with the value of FIRE [Figs. 10 ▸(*a*4)–10 ▸(*c*4)]. The radiation dose for *N*_p_ = 500 is lower than for the two other cases (see Table 1). Therefore, *N*_p_ = 500 is the optimum when considering image quality and radiation dose.

### 3.5. Low-dose imaging

**Figure 11:**
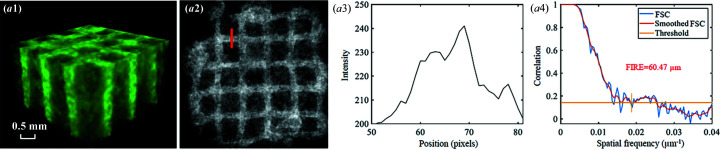
Reconstruction results of a hydro­gel scaffold using SR-PBI-HCT with *N*_p_ = 250 (2 × 2 binning): (*a*1) reconstructed 3D result; (*a*2) corresponding ROI image; (*a*3) corresponding gray value profile measured at the red line positions in (*a*2); (*a*4) 3D spatial resolution analysis with FSC, smoothed FSC and FIRE.

Although the image quality is satisfactory with *E* = 30 keV and *N*_p_ = 500, the 17.67 Gy radiation dose is high for *in vivo* live-animal imaging. We tested the feasibility of SR-PBI-HCT for hydro­gel scaffold imaging within an acceptable dose range [∼500 mGy for rat protocols (Pratt *et al.*, 2014 ▸)]. Specifically, a scaffold imaging experiment with 2 × 2 binning mode and *N*_p_ = 250 was also conducted. In addition, an NDF with a thickness of 80 mm was used to reduce the photon flux. The measured radiation dose was 54.72 mGy s^−1^. Results (Fig. 11 ▸) show that scaffolds can still be identified and segmented from the background (*i.e.* water) while the radiation dose (∼342 mGy) remains suitable for *in vivo* animal imaging. This study reveals that SR-PBI-HCT imaging, though lacking *in vivo* results currently, is promising for *in vivo* TERM applications.

## 4. Conclusions

In TERM, visualization of low-density tissue scaffolds following their implantation is crucial, yet challenging. In this paper, we present a study on the integration of helical acquisition mode with SR-PBI-CT to non-invasively and non-destructively visualize/characterize low-density scaffolds. The results demonstrate the improvement of contrast and the significant advantage of avoiding ring artifacts without introducing additional artifacts. We also showed that *p* = 1.5, *E* = 30 keV and *N*_p_ = 500 were suited for hydro­gel scaffold imaging *in vitro* using the current SR-PBI-HCT configuration of BMIT. In addition, with 2 × 2 binning mode and *N*_p_ = 250, the SR-PBI-HCT can produce satisfactory results while the radiation dose (∼342 mGy, voxel size of 26 µm) remains suitable for *in vivo* animal imaging. The results obtained in this study reveal that the SR-PBI-HCT imaging method is a powerful tool for visualizing and characterizing hydro­gel scaffolds in terms of image quality and radiation dose, forming a solid base for *in vivo* 3D non-invasive characterization in TERM.

## Supplementary Material

S1: why 1.5 m was used for SR-PBI-CT/HCT imaging. S2: details of the required scanning angle for SR-PBI-HCT. DOI: 10.1107/S1600577523000772/tv5040sup1.pdf

## Figures and Tables

**Table 1:** Experimental details in terms of imaging conditions for five different group tests

Group	Scan mode	Imaged object	Energy (keV)	Projection number (rev^−1^)	Binning mode	Exposure time (ms per projection)	Helical pitch	Measured dose (Gy)
No. 1: SR-PBI-CT versus SR-PBI-HCT	SR-PBI-CT	Hydro­gel scaffold (4% *w*/*v* alginate)	30	3000	1 × 1	57.00	–	79.51
		3D QRM bar pattern phantom						
	SR-PBI-HCT	Hydro­gel scaffold (4% *w*/*v* alginate)	30	3000	1 × 1	57.00	1.3	122.33
		3D QRM bar pattern phantom						
No. 2: Helical pitch comparison	SR-PBI-HCT	Hydro­gel scaffold (3% *w*/*v* alginate with 1% *w*/*v* gelatin)	30	3000	1 × 1	57.00	1.3	122.33
							1.5	106.02
							1.7	93.54
							1.9	83.70
							2.5	63.61
No. 3: Photon energy comparison	SR-PBI-HCT	Hydro­gel scaffolds (4% *w*/*v* alginate)	30	3000	1 × 1	57.00	1.5	106.02
			40			14.40		48.38
			50			10.82		31.16
No. 4: Projection number per revolution comparison	SR-PBI-HCT	Hydro­gel scaffold (3% *w*/*v* alginate with 1% *w*/*v* gelatin)	30	500	1 × 1	57.00	1.5	17.67
				1000				35.34
				3000				106.02
No. 5: Low-dose imaging	SR-PBI-HCT	Hydro­gel scaffold (3% *w*/*v* alginate with 1% *w*/*v* gelatin)	30	250	2 × 2	25.00	1.5	0.34
