# Supplementary material for: Low-density tissue scaffold imaging by synchrotron radiation propagation-based imaging computed tomography with helical acquisition mode
Source: J Synchrotron Radiat. 2023 Feb 16;30(Pt 2):417–29. doi: 10.1107/S1600577523000772 (PMC10000810; doi:10.1107/S1600577523000772)
Supplement: Supplementary file 1 [file s-30-00417-sup1.pdf]

JOURNAL OF SYNCHROTRON RADIATION

Volume 30 (2023)

Supporting information for article:

Low-density tissue scaffold imaging by synchrotron radiation propagation-based imaging computed tomography with helical acquisition mode

Xiaoman Duan, Naitao Li, David M. L. Cooper, Xiao Fan Ding, Xiongbiao Chen and Ning Zhu

Section S1

A hydrogel scaffold was scanned *in vitro* at BMIT-ID (CLS) with SDD of 0.22 m, 0.55 m, 1.0 m, 1.5 m, and 2.0 m, respectively. The main influences of SDD on imaging is the spatial resolution for image quality and/or phase shifts for phase contrast imaging.

Spatial resolution can be reflected by the strand's edge sharpness which can be measured by edge grey value profiles. The grey value profiles of edge were tested for images obtained using varied SDD [Fig. S1 (A2-E2)] before phase retrieval. Results show 1.5 m can produce highest sharpness for edges. Phase shifts can be reflected by the contrast-to-noise ratio (CNR, see Section 2.4 in the manuscript) index after presenting phase retrieval with same parameters. CNR was measured for results obtained using varied SDD [Fig. S1 (A3-E3)]. Results show images acquired at 1.5 m can produce highest CNR (i.e., 6.29), compared with the four other images.

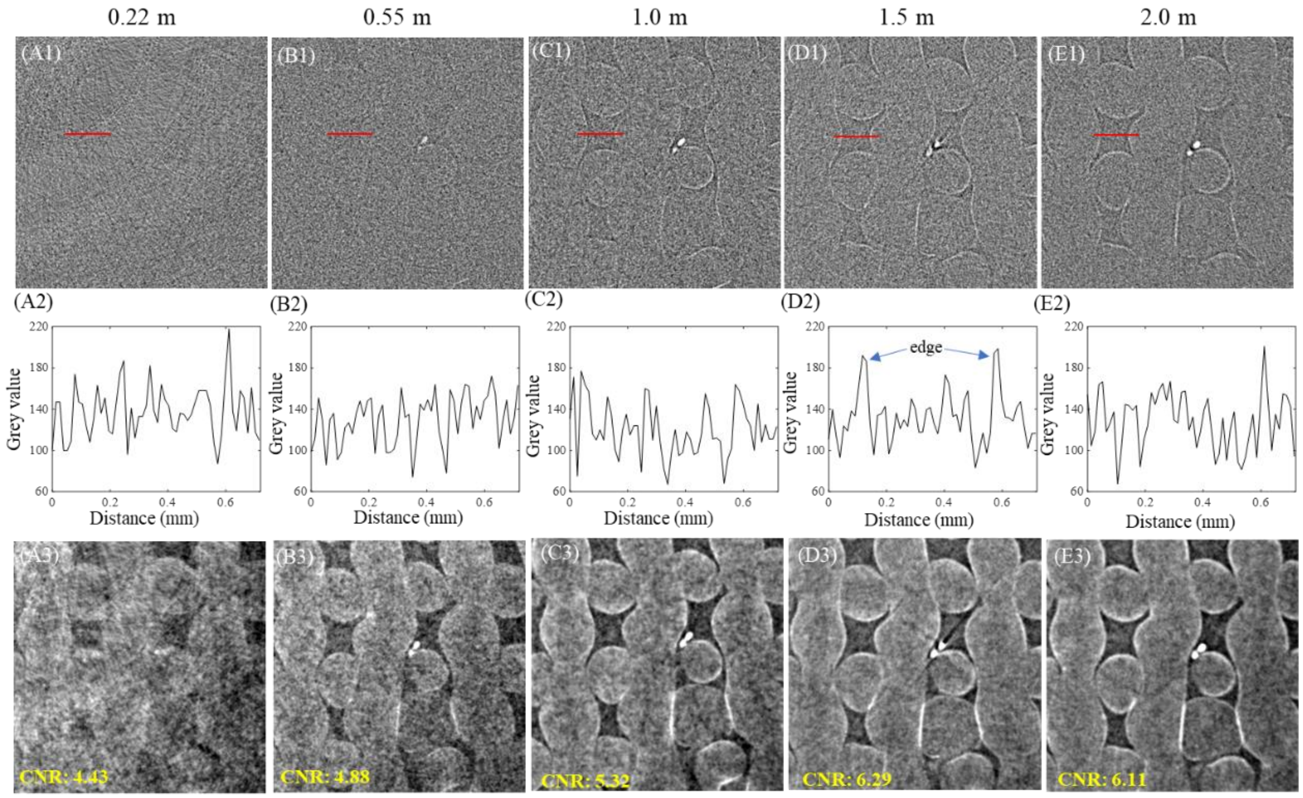

Figure S1 Effect of sample-to-detector distance (SDD, i.e., 0.22 m, 0.55 m, 1.0 m, 1.5 m, and 2.0 m) on hydrogel scaffold images using SR-PBI-CT. (A1-E1) reconstructed results before phase retrieval; (A2-E2) profiles of strand's edge (red lines) before phase retrieval; (A3-E3) reconstructed results after phase retrieval.

Section S2

Below we provide some details to explain the required scanning angle of SR-PBI-HCT.

For each voxel in reconstruction, the data (i.e., projections) acquired across 180° is necessary and sufficient (for parallel beam). This is applicable to both SR-PBI-CT and SR-PBI-HCT. The following scanning illustration of relative positions between the region of interest (ROI) and the detector at different angles can help one to understand the required scanning angle for SR-PBI-HCT (taking pitch of 2 as an example).

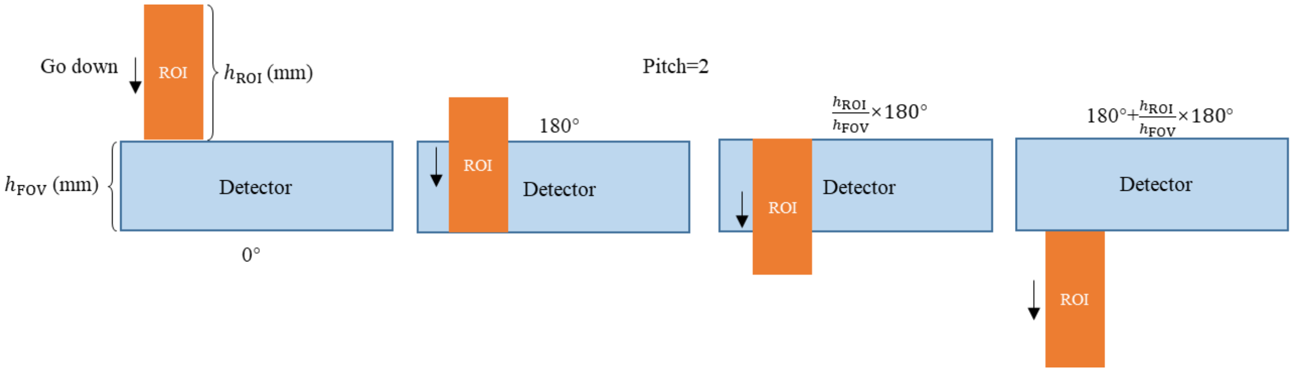

Figure S2 Scanning illustration of relative positions between the ROI and the detector.

The h_{ROI} (unit of mm) is the height of the ROI and the rotation stage moves down during the scan. The detector remains stationary during the scan and the height of the field of view (FOV) (i.e., the height of the active detector) is h_{FOV} (unit of mm). When the bottom of the ROI just enters the FOV, that sample position is considered as the scanning angle 0°. On this basis, when the bottom of the ROI just leaves the FOV, the acquisition angle will be exactly 180° in the condition of the pitch of 2. The data are sufficient for completing reconstruction. The angle when the top of the ROI just enters the FOV can be calculated as $\frac{h_{ROI}}{h_{\mathrm{FOV}}} \times 180°$. When the top of the ROI just leaves the FOV, the acquisition angle will be $180° + \frac{h_{ROI}}{h_{\mathrm{FOV}}} \times 180°$. As a result, for ROI, the acquisition angle will be from 0° to $180° + \frac{h_{ROI}}{h_{\mathrm{FOV}}} \times 180°$. The travelling acquisition angle is determined by h_{ROI}, h_{FOV}, and the pitch. For the pitch of 2, when the height of the ROI is the same as the FOV height (i.e., $h_{ROI} = h_{\mathrm{FOV}}$), data acquired in 360° rotation is required; when the ROI is twice high as the FOV, (i.e., $h_{ROI} = 2h_{\mathrm{FOV}}$), the scan needs to be done in 540°; and 720° scans work for the condition of $h_{ROI} = 3h_{\mathrm{FOV}}$. By using SR-PBI-HCT, usually the sample ROI is higher than the detector FOV ($h_{ROI} > h_{\mathrm{FOV}}$) and that's the reason we claim the SR-PBI-HCT usually involves the scan rotation over 360°.
